# The complete mitogenome of excavating sponge *Thoosa mismalolli* (Demospongiae, Tetractinellida, Thoosidae) from Northeastern Tropical Pacific

**DOI:** 10.1080/23802359.2020.1714499

**Published:** 2020-01-16

**Authors:** Eric Bautista-Guerrero, Raúl Llera-Herrera, José L. Carballo, Axayácatl Rocha-Olivares, Alma P. Rodríguez-Troncoso, Adrian González-Castillo, José A. Cruz-Barraza

**Affiliations:** a Laboratorio de Ecología Marina, Centro de Investigaciones Costeras, Centro Universitario de la Costa, Universidad de Guadalajara, Puerto Vallarta, Mexico;; b Instituto de Ciencias del Mar y Limnología, Universidad Nacional Autónoma de México (Unidad Académica Mazatlán), Mazatlán, Mexico;; c Molecular Ecology Laboratory, Biological Oceanography Department, CICESE, Ensenada, Mexico

**Keywords:** Porifera, genus *Thoosa*, mitogenome, excavating sponge, Eastern Tropical Pacific

## Abstract

The complete mitogenome of *Thoosa mismalolli* Carballo, Cruz-Barraza & Gómez, 2004 (Tetractinellida, Thoosidae) was sequenced. This is the first complete mitogenome of the suborden Thoosina and the third Tetractinellid so far. The mitochondrial genome of *T. mismalolli* was assembled based on reads obtained with the Illumina HiSeq platform. The length of complete mitogenome is 19,019 bp long and contained 14 protein-coding genes and 23 tRNA, with two tRNA genes. Phylogenetic reconstruction (maximum-likelihood) based on mitogenome of Tetractinellids, supports *T. mismalolli* as a sister group. This result is congruent with those obtained with molecular markers (CO1, 18S, and 28S), supporting the monophyletic status of *Thoosa* and providing additional molecular data in favor of the suborder Thoosina.

## Introduction

Marine sponges are an abundant and diverse benthic groups that perform relevant functional roles in the marine ecosystems (Bell 2008). Boring sponges of genus *Thoosa* are important because they excavate calcareous substrate, through chemical and physical mechanisms, and therefore contribute with the carbonate budget in these ecosystems (Pomponi 1977; Nava and Carballo 2008). At the Northeastern Tropical Pacific (NTP), three species of *Thoosa* have been recorded. *Thoosa mismalolli* is widespread distributed along the NTP reef communities where is one of the most conspicuous and aggressive bioeroder (Carballo et al. 2013). Reproductive biology and ecology of this species is well known (Bautista-Guerrero et al. 2016), however, there is no knowledge of its mitogenome which is fundamental to assess the population genetics and its evolutionary biology. This study describes the mitogenome of *T. mismalolli*, and their phylogenetic relationships with other tetractinellids.

Samples of *T. mismalolli* invading pocilloporid coral, were collected at Isabel Island, Mexico (21.52°N, 105.54°W). Specimens (voucher: LEB-ICML-UNAM-3179) were deposited in the sponge collection (OAX-MAM-135-10-02) at the Instituto de Ciencias del Mar y Limnología-UNAM. Genomic DNA was extracted using SV Promega kit following the manufacturer’s instruction. Genomic library was prepared using the Nextera^®^ XT DNA library kits, and sequenced with the Illumina HiSeq platform. A total of 15’082,838 paired-reads were obtained, and processed with Trimmomatic (Bolger et al. 2014). Only 13,107,511 paired-reads with an average Qscore of 37.9 were retained, and assembled *de novo* using Megahit v1.2.2-beta (Li et al. 2016) and local BLAST. The overlapping ends were trimmed, and the starting position was re-oriented using the mitogenome of *Poecillastra laminaris* (NCBI: KM362735.1). The annotation was performed in MITOS web server (Bernt et al. 2013).

The complete mitogenome of *T. mismalolli* consist of 19,019 bp (GenBank: MN587873) and included 14 protein-coding genes, 23 tRNA genes and two rRNA subunits. The nucleotide composition is A (29.9%), T (36.8%), C (12.8%) and G (20.4%) with a GC content of 33.2%. The arrangement of protein in the mitogenome of *T. mismalolli* was similar to the rest of tetractinellid sponges; however, it is important to emphasize that it resulted 4.8% longer than *Pocillastra laminaris*, ∼3.18% than both *Geodia neptuni* and *Cinachyrella kuekenthali* (Lavrov et al. 2005, 2008; Zeng et al. 2016). This change in the mitogenome size is essentially due to insertions in non-coding regions.

**Figure 1.**
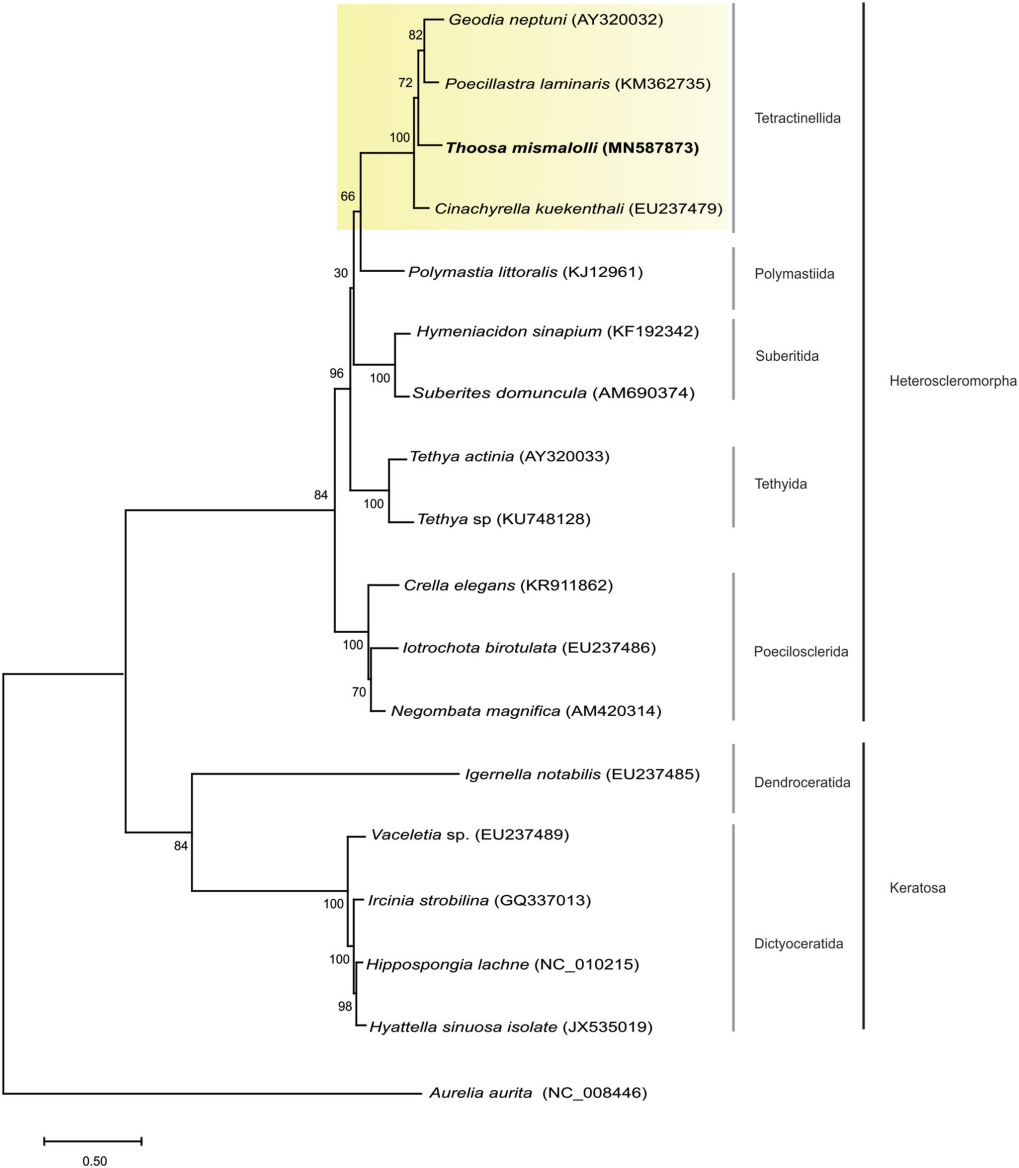
Consensus tree of *Thoosa mismalolli* using Maximum-Likelihood (ML) method based on complete mitogenomes of species belonging to subclass Heteroscleromorpha and Verongimorpha. Bootstrap values are indicated above the principal node with 1000 bootstrap replicates. Taxon names are followed by their Genbank accession number. Yellow box indicates the Tetractinellida Order.

Phylogenetic analysis was performed using MEGA7^®^ software to reconstruct ML tree (Tamura et al. 2013). GTR + G resulted as the best-fitting nucleotide substitution model according to the JModelTest 2.0 Software (Posada 2008). *Aurelia aurita* (NC_008446.1) was used as outgroup. Phylogenetic reconstruction (Figure 1) indicated that *T. mismalolli* is monoplyletic and formed a strongly supported clade with the sister Tetractinellida group. Overall, relationship was congruent with earlier findings on individual mitochondrial (CO1 mtDNA) and ribosomal (28S and 18S rRNA) markers as well as morphological data (Carballo et al. 2018), confirming the monophyletic status of *Thoosa* and supporting the recent creation of new suborder (Thoosina) of Tetractinellida proposed by Carballo et al. (2018).
